# Molecular phenotypes and clinical characterization of familial hereditary breast cancer among half and full sisters

**DOI:** 10.1186/s12905-022-01732-y

**Published:** 2022-05-02

**Authors:** Yingjie Xu, Jun He, Chen Qian, Chengguang Yang

**Affiliations:** Department of General Surgery, Tongren Hospital, Shanghai Jiao Tong University School of Medicine, 1111 XianXia Road, Shanghai, 200336 China

**Keywords:** Breast cancer, Hereditary factors, Molecular phenotype, Modified radical mastectomy, Pathological characteristics

## Abstract

**Background:**

Preliminary clinical observations show that contemporaneous hereditary breast cancer (CHBC) patients suffered breast cancer at an early age, which requires further analysis.

**Methods:**

38 familial hereditary breast cancer patients (18 CHBC patients and 20 non-CHBC patients) were screened out and 152 non-hereditary breast cancer patients were used as control subjects. Clinical pathologic subtypes, age, tumor location, histological grade, lymph node metastasis, and molecular phenotype expression (*ER, PR, HER-2, Ki-67, CK5/6, E-cad, P63,* and *P120*) were compared across all subgroups.

**Results:**

The incidence of CHBC was 9.47% (18/190) in breast cancer patients. The average ages of onset of CHBC patients, non-CHBC patients, and non-hereditary breast cancer patients were 49.06 ± 6.42, 60.75 ± 9.95 and 61.69 ± 14.34 respectively; whereas there were no significant differences with respect to pathological type or tumor location. There were significant differences in some histological grading (grade II/III), lymph node metastasis and *PR* expression between hereditary and non-hereditary breast cancers (*P* < 0.05; *P* < 0.05 and *P* < 0.005, respectively). Significantly different *HER-2* expression was observed when comparing all hereditary or CHBC patients with non-hereditary breast cancers (*P* < 0.05 and *P* < 0.005, respectively). There were significant differences in *E-cad* and *P63* between contemporaneous hereditary and non-hereditary breast cancers (*P* < 0.005 and *P* < 0.05, respectively).

**Conclusions:**

CHBC patients accounted for 9.47% (18/190) of breast cancer patients, had earlier disease onset, and showed differences compared to non-hereditary breast cancer patients with respect to molecular phenotype and clinical characteristics.

## Introduction

Recently, the incidence of breast cancer in female patients has increased each year, and hereditary factors play an important role in the risk of breast cancer. Clinical observation and literature reports show that breast cancer with a hereditary family background often has an earlier onset. Many studies have studied the genetic factors of breast cancer [1–3], which are often accompanied by hereditary gene mutations [3–5]. However, there are few reports on the incidence characteristics of some particular subgroups of hereditary breast cancer, including patients with contemporaneous hereditary breast cancer (CHBC) background; furthermore, breast cancer diagnosis and treatment guidelines rarely consider this subgroup [6, 7]. Preliminary clinical observations suggest that breast cancer patients with contemporaneous hereditary backgrounds develop cancer earlier, which may progress more rapidly. Therefore, further studies are necessary for this population. In this study, we retrospectively analyzed hereditary breast cancer patients (with contemporaneous hereditary and non-contemporaneous genetic backgrounds) and non-hereditary breast cancer patients concerning clinical pathogenesis type, age, tumor location, histological grade, lymph node metastasis, and possible differences in molecular phenotype expression. Here, we explored the clinical characteristics and molecular phenotypes of contemporaneous hereditary breast cancer patients to provide clinical references and a basis for studying the hereditary factors in breast cancer.

## Patients and methods

**Fig. 1:**
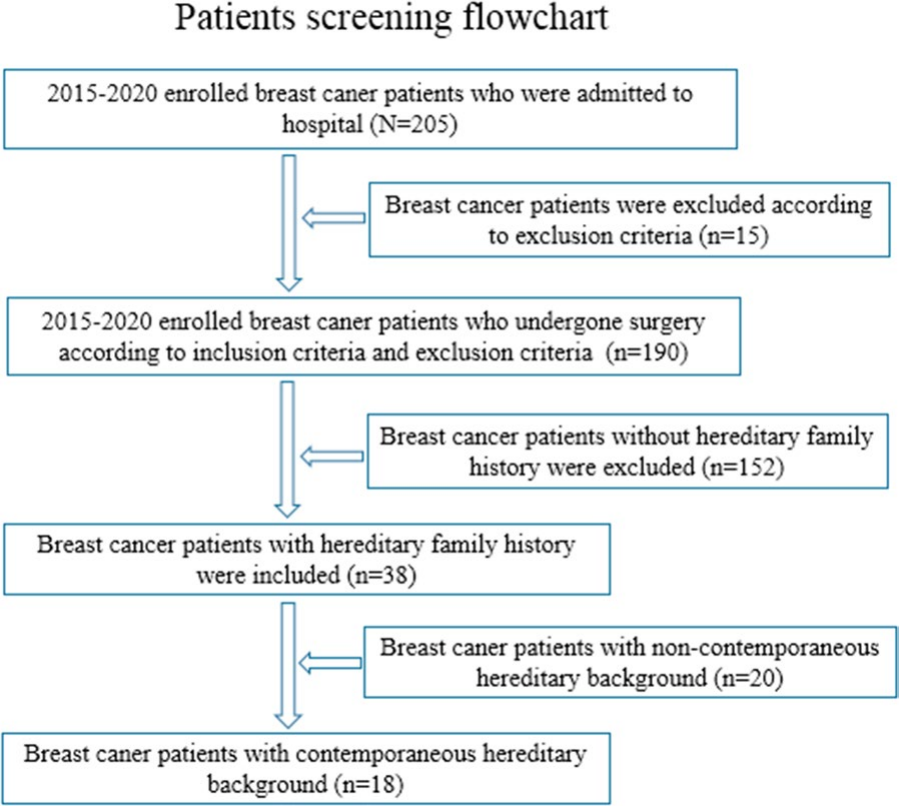
Breast cancer patients screening flow chart

A retrospective analysis was conducted involving 190 female patients who received modified radical mastectomy for breast cancer in Shanghai Tongren Hospital from January 2015 to December 2020; 38 breast cancer patients with hereditary family backgrounds were included (including 18 contemporaneous hereditary backgrounds and 20 non-contemporaneous hereditary background) and 152 non-hereditary background breast cancer patients treated during the same period were used as control subjects (see Fig. 1). Clinical pathological subtypes, age, tumor location, histological grade, lymph node metastasis, and molecular phenotype expression (*ER, PR, HER-2, Ki-67, CK5/6, E-cad, P63,* and *P120*) were compared across all subgroups in the present study. A detailed medical history was obtained for each patient, and written informed consent preoperatively, the study protocol was approved by the Shanghai Tongren hospital ethics committee. Pathological diagnosis of breast cancer and pathological features were independently reviewed by three experienced pathologists using the intraoperative frozen sections and postoperative paraffin sections. The clinical characteristics of patients with contemporaneous hereditary, non-contemporaneous hereditary, and non-hereditary breast cancer are shown in Table 1.

**Table 1:** Demographic characteristics of breast cancer patients

Variable	NHBC	HBC			Total
		CHBC	NCHBC	CHBC + NCHBC	
Patients, n	152	18	20	38	190
Ages, years	61.69 ± 14.34	49.06 ± 6.42	60.75 ± 9.95	55.21 ± 10.24	60.41 ± 13.85
Anatomical site					
Left	90 (59.21%)	11 (61.11%)	8 (40%)	19 (50%)	109 (57.37%)
Right	62 (40.79%)	7 (38.89%)	12 (60%)	19 (50%)	81 (42.63%)
Pathologic types, n					
Invasive ductal carcinoma	116 (76.32%)	11 (61.11%)	15 (75%)	26 (68.42%)	142 (74.74%)
Ductal carcinoma in situ	21 (13.82%)	6 (33.33%)	4 (20%)	10 (26.32%)	31 (16.32%)
Mucinous carcinoma	8 (5.26%)	0	1 (5%)	1 (2.63%)	9 (4.74%)
Intraductal papillary carcinoma	5 (3.29%)	1 (5.56%)	0	1 (2.63%)	6 (3.16%)
Medullary carcinoma	2 (1.31%)	0	0	0	2 (1.04%)
Cell differentiation, n					
Grade I/high-level	11 (7.24%)	3 (16.66%)	1 (5%)	4 (10.53%)	15 (7.89%)
Grade II/middle-level	89 (58.55%)	12 (66.67%)	9 (45%)	21 (55.26%)	110 (57.89%)
Grade III/low-level	52 (34.21%)	3 (16.66%)	10 (50%)	13 (34.21%)	65 (34.22%)
Pathological stages	T_0–3_N_0–3_M_0_	T_0–3_N_0–3_M_0_	T_0–3_N_0–3_M_0_		
Lymph node metastasis, n					
Positive	51 (33.55%)	10 (55.55%)	10 (50%)	20 (52.63%)	71 (37.37%)
Negative	101 (66.45%)	8 (44.45%)	10 (50%)	18 (47.37%)	119 (62.63%)

Continuous variables are presented as mean ± standard deviation, categorical variables are presented as numbers (percentages)

*HBC* hereditary breast cancer, *NHBC* non-hereditary breast cancer, *CHBC* contemporaneous hereditary breast cancer, *NCHBC* non-contemporaneous hereditary breast cancer

*Inclusion criteria* Patients with a primary breast cancer diagnosis who underwent modified radical breast cancer surgery were included in the study.

*Exclusion criteria* Patients with a primary diagnosis of breast cancer but could not undergo modified radical breast cancer surgery due to the following reasons: (1) patients with severe heart, lung, and kidney dysfunction upon preoperative examination; (2) patients taking oral aspirin or other anticoagulant drugs; (3) patients with abnormal coagulation found during the preoperative examination; (4) patients who refused surgery after admission.

### Contemporaneous hereditary and non-contemporaneous hereditary breast cancer definitions

Contemporaneous hereditary breast cancer (CHBC) patients are half-sisters or full sisters who have the same mother and/or father and whose parents or elder relatives have also had breast cancer. Non-contemporaneous hereditary breast cancer (NCHBC) patients are not half-sisters or full sisters but those with parents or elder relatives who have had breast cancer. No male breast cancer patients were observed in this study.

### Histological grade description

Invasive breast cancer was classified as grade I (highly differentiated), grade II (moderately differentiated), or grade III (poorly differentiated) upon evaluation of morphological features (tubulography, nuclear pleomorphism, and calibrated mitotic counts) based on the Nottingham Combined Histological Grading criteria (modified Scarff-Bloom-Richardson Grading System) [8]. Breast cancer in situ was classified as low grade (grade I), medium grade (grade II), or high grade (grade III) according to mitotic counts.

### Statistical analysis

Statistical analysis was performed using Origin 8.0 software (Origin Lab Corp. Northampton, MA, USA). Continuous variables are presented as mean ± standard deviation and were compared using the student’s *t* test. Categorical variables are presented as numbers (percentages) and were compared using the Chi-squared test or Fisher’s exact test. *P* < 0.05 was used as the threshold for statistical significance.

## Results

### Patient demographic characteristics

Demographic characteristics were analyzed in 190 hereditary and non-hereditary breast cancer patients who underwent modified radical breast cancer surgery (Table 1). The incidence was 20% (38/190) for familial hereditary breast cancer, and 9.47% (18/190) for CHBC. All breast cancer patients enrolled in this study were female. The average age of onset of breast cancer was 49.06 ± 6.42 years in CHBC patients and 60.75 ± 9.95 (years) in NCHBC patients, while that of non-hereditary breast cancer patients was 61.69 ± 14.34 years. The incidence of hereditary breast cancer in the left side was similar to that observed in the right side (50%, 19/38), while the incidence of non-hereditary breast cancer was slightly higher in the left (59.21%, 90/152). Patients were included in this study with pathological stage T_0-3_N_0-3_M_0_. Among the pathological types of breast cancer, the incidence rate of invasive ductal carcinoma was the highest (74.74%, 142/190), followed successively by ductal carcinoma in situ (16.32%, 31/190), mucinous carcinoma (4.74%, 9/190), intraductal papillary carcinoma (3.16%, 6/190), and medullary carcinoma (1.04%, 2/190). The proportions of various pathologic types in hereditary and non-hereditary breast cancers were similar (Table 1). Lymph node metastasis was found in 52.63% (20/38) of hereditary breast cancer patients and 33.55% (51/152) of non-hereditary breast cancer patients in the surgical population.

### Comparison of age of onset age across breast cancer subgroups

In this study, differences in the age of onset age of hereditary and non-hereditary breast cancer were compared. The results show (Table 2) that the average age of onset of breast cancer was 60.41 ± 13.85 years in the surgical population, 61.69 ± 14.34 years for non-hereditary breast cancer patients, and 49.06 ± 6.42 years for hereditary breast cancer patients. The mean age of the onset of hereditary breast cancer was 60.75 ± 9.95 years. Among the subgroups, significant differences were found between CHBC patients and NCHBC patients with respect to mean age of breast cancer onset; significant differences were also observed between whole hereditary breast cancer patients (CHBC patients and NCHBC patients) and non-hereditary breast cancer patients (*t* = 4.25, *P* < 0.001, *t* = 2.34, *P* < 0.05, *t* = 3.41, *P* < 0.001, respectively). Significant differences were also observed between NCHBC patients and whole breast cancer patients with respect to average age of breast cancer onset (*t* = 2.15, *P* < 0.05), and among hereditary breast cancer patients, non-hereditary breast cancer patients, and whole breast cancer patients with respect to average age of the breast cancer onset (*t* = 2.57, *P* < 0.05; *t* = 2.15, *P* < 0.05, respectively). There were no significant differences among other subgroups. The age of onset for hereditary breast cancer patients was significantly earlier than that of the whole breast cancer patients, especially in the contemporaneous hereditary group (*P* < 0.001).

**Table 2:** Comparison of age of onset age across breast cancer subgroups

Variable	NHBC	HBC			Total	
		CHBC	NCHBC	CHBC + NCHBC		
Patients, n	152	18	20	38	190	
Ages, years	61.69 ± 14.34	49.06 ± 6.42	60.75 ± 9.95	55.21 ± 10.24	60.41 ± 13.85	
	*t* = 2.57 *P *< 0.05	*t* = 4.25 *P < 0.01	*t* = 0.25 **P > 0.05	*t* = 4.25 ***P < 0.01	*t* = 3.4 ^#^*P *< 0.001	*t* = 2.15 ^△^*P *< 0.05

Continuous variables are presented as mean ± standard deviation

*HBC* hereditary breast cancer, *NHBC* non-hereditary breast cancer, *CHBC* contemporaneous hereditary breast cancer, *NCHBC* non-contemporaneous hereditary breast cancer

*P* = NHBC versus HBC, *P = NHBC versus CHBC, **P = NHBC versus NCHBC, ***P = CHBC versus NCHBC, ^#^*P* = CHBC versus (NHBC + HBC), ^△^*P* = NCHBC versus (NHBC + HBC)

### Comparison of lesion location across breast cancer subgroups

The incidence of breast cancer patients with different backgrounds in left and right anatomic sites was compared. The results show that there were no significant differences between hereditary breast cancer patients and non-hereditary breast cancer patients with respect to anatomic sites (*X*^2^ = 1.05, *P* > 0.05). Likewise, there were also no significant differences among the CHBC patients, NCHBC patients, and non-hereditary breast cancer patients with respect to anatomic sites (*X*^2^ = 1.69, *P* > 0.05, *X*^2^ = 0.02, *P* > 0.05, respectively). Furthermore, there were no significant differences between NCHBC patients and non-hereditary breast cancer patients with respect to anatomic sites (*X*^2^ = 2.66, *P* > 0.05). Comprehensive comparison results among all subgroups showed that there were no statistical differences in the incidence of bilateral anatomic sites of breast cancer under different backgrounds (*P* > 0.05).

### Comparison of pathological types and cell differentiation grades across breast cancer subgroups

Table 1 shows that the incidence rate of invasive ductal carcinoma was highest (74.74%, 142/190) in the population that underwent modified radical breast cancer surgery, followed successively by ductal carcinoma in situ (16.32%, 31/190), mucinous carcinoma (4.74%, 9/190), intraductal papillary carcinoma (3.16%, 6/190), and medullary carcinoma (1.04%, 2/190). The proportions were similar for various pathological types of hereditary and non-hereditary breast cancer (Table 1). Among hereditary breast cancers, the incidence rate of invasive ductal carcinoma was highest (68.42%, 26/38), followed successively by ductal carcinoma in situ (26.32%, 10/38), mucinous carcinoma (2.63%, 1/38), and intraductal papillary carcinoma (2.63%, 1/38); the incidence rate of invasive ductal carcinoma was also the highest (61.11%, 11/18) in the CHBC, followed successively by ductal carcinoma in situ (33.33%, 6/18) and intraductal papillary carcinoma (5.56%, 1/18). There were no significant differences in the proportion of pathological types among the other subgroups (*P* > 0.05; Table 3). Cell differentiation grading comparison results showed that grade II (moderate differentiation/middle level) accounted for the highest percentage (57.89%, 110/190), followed by grade III (poor differentiation/low level; 34.22%, 65/190), with grade I (high differentiation/high level) accounting for the lowest (7.89%, 15/190). The cell differentiation grade proportion of hereditary and non-hereditary breast cancer was similar (Table 1). In hereditary breast cancer, the incidence rate of grade II was the highest (55.26%, 21/38), followed by grade III (34.21%, 13/38), grade I was the lowest (10.53%, 4/38). The incidence rate of grade II was also the highest (66.67%, 12/18) in CHBC. However, the proportion of grade III and grade I was the same in each subgroup (16.66%, 3/18, respectively). There were no significant differences with respect to cell differentiation grade proportion across the other subgroups (*P* > 0.05; Table 3).

**Table 3:** Comparison of pathological types and cell differentiation grades across breast cancer subgroups

Variable	NHBC	HBC			Total
		CHBC	NCHBC	CHBC + NCHBC	
Patients, n	152	18	20	38	190
Pathologic types, n					
Invasive ductal carcinoma	116	11	15	26	142
Ductal carcinoma in situ	21	6	4	10	31
Mucinous carcinoma	8	0	1	1	9
Intraductal papillary carcinoma	5	1	0	1	6
Medullary carcinoma	2	0	0	0	2
	*X*^2^ = 3.89 *P *> 0.05	*X*^2^ = 0.74 *P > 0.05	*X*^2^ = 4.69 **P > 0.05	*X*^2^ = 0.85 ^*#*^*P *> 0.05	
Cell differentiation, n					
Grade I/high-level	11	3	1	4	15
Grade II/middle-level	89	12	9	21	110
Grade III/low-level	52	3	10	13	65
	*X*^2^ = 0.48 *P *> 0.05	*X*^2^ = 3.43 *P > 0.05	*X*^2^ = 1.91 **P > 0.05	*X*^2^ = 5.11 ^*#*^*P *> 0.05	

*HBC* hereditary breast cancer, *NHBC* non-hereditary breast cancer, *CHBC* contemporaneous hereditary breast cancer, *NCHBC* non-contemporaneous hereditary breast cancer

*P* = NHBC versus HBC, *P = NHBC versus CHBC, **P = NHBC versus NCHBC, ^#^*P* = CHBC versus NCHBC

### Comparison of breast cancer with lymph node metastasis across subgroups

In this study, breast cancer patients with lymph node metastasis accounted for 37.37% (71/190) of the surgery population (Table 1), 33.35% (51/152) of the non-hereditary breast cancer population, 52.63% (20/38) of the hereditary breast cancer population, 55.55% (10/18) of the CHBC population, and 50% (10/20) of the NCHBC population. Pairwise comparison among subgroups showed that there was a significant difference in the proportion of patients with lymph node metastasis only between hereditary and non-hereditary breast cancer subgroups (*X*^2^ = 4.73, *P* < 0.05; Table 4). There were no significant differences in the proportion of patients with lymph node metastasis among other subgroups (*P* > 0.05; Table 4).

**Table 4:** Comparison of breast cancer with lymph node metastasis across subgroups

Variable	NHBC	HBC			Total
		CHBC	NCHBC	CHBC + NCHBC	
Patients, n	152	18	20	38	190
Lymph node metastasis, n					
Positive	51	10	10	20	71
Negative	101	8	10	18	119
	*X*^2^ = 4.73 *P *< 0.05	*X*^2^ = 3.39 *P > 0.05	*X*^2^ = 2.09 **P > 0.05	*X*^2^ = 0.12 ^#^*P *> 0.05	

*HBC* hereditary breast cancer, *NHBC* non-hereditary breast cancer, *CHBC* contemporaneous hereditary breast cancer, *NCHBC* non-contemporaneous hereditary breast cancer

*P* = NHBC versus HBC, *P = NHBC versus CHBC, **P = NHBC versus NCHBC, ^#^*P* = CHBC versus NCHBC

### Expression of *estrogen receptor* (*ER*), *progesterone receptor* (*PR*), *human epidermal growth factor receptor 2* (*HER-2*), and *Ki-67* across the breast cancer subgroups

Related molecular markers of breast cancer were observed in this study, and the expression levels of various molecular markers (including *ER, PR, HER-2,* and *Ki-67*) are shown in Table 5. For *ER*, a comparative study among the subgroups found no significant differences with respect to *ER* marker expression in breast cancer with different backgrounds (*P* > 0.05; Table 6). There were significant differences in the expression level of *PR* between hereditary and non-hereditary breast cancer patients (*X*^*2*^ = 13.38,* P* < 0.005), and in the expression level of *PR* (++) and *PR* (+++) (*X*^*2 *^= 6.11, *P* < 0.05). There was also a significant difference in the expression level of *PR* between CHBC and non-hereditary breast cancer patients (*P* < 0.05), not only with respect to the expression level of *PR* (++) (*X*^2 ^= 6.42, *P* < 0.05), but also for the expression level of *PR* (+++) (*X*^*2*^ = 4.66, *P* < 0.05). There were no significant differences in the expression level of *PR* across the other subgroups (*P* > 0.05; Table 6). There was a significant difference in the expression level of *HER-2* between hereditary and non-hereditary breast cancer patients (*X*^2 ^= 7.96, *P* < 0.05; Table 7), specifically with respect to the expression level of *HER-2* (+++) (*X*^2 ^= 7.24, *P* < 0.01). Moreover, a significant difference in the expression level of *HER-2* was also observed between CHBC and non-hereditary breast cancer patients (*X*^2 ^= 11.55, *P* < 0.005), specifically with respect to the expression level of *HER-2* (+++) (*X*^2 ^= 11.56, *P* < 0.001). There were no significant differences in *HER-2* expression across the other subgroups (*P* > 0.05; Table 7). There were also no significant differences in the expression levels of *Ki-67* in the breast cancer subgroups with different backgrounds (*P* > 0.05; Table 7).

**Table 5:** Enzyme-labeled staining for *ER*, *PR*, *HER*-*2* and *Ki*-*67* across subgroups

Variable	NHBC	HBC			Total
		CHBC	NCHBC	CHBC + NCHBC	
Expression of *ER*, n	152	18	20	38	190
(−)	44	2	2	4	48
(+)	22	1	5	6	28
(++)	15	7	1	8	23
(+++)	71	8	12	20	91
Expression of *PR*, n					
(−)	53	2	0	2	55
(+)	25	1	8	9	34
(++)	28	5	7	12	40
(+++)	46	10	5	15	61
Expression of *HER-2*, n					
(−)	66	2	6	8	74
(±) (fish negative)	22	6	5	11	33
(+) (fish positive)	16	2	3	5	21
(++)	40	3	4	7	47
(+++)	8	5	2	7	15
Expression of *Ki-67*, n					
(−)	36	6	7	13	49
(+)	36	2	3	5	41
(++)	60	6	8	14	74
(+++)	20	4	12	6	26
Molecular phenotypes, n					
Luminal A	51	5	10	15	66
Luminal B	10	3	1	4	14
Her-2(+)	76	10	9	10	95
Triple negative	15	0	0	0	15

*HBC* hereditary breast cancer, *NHBC* non-hereditary breast cancer, *CHBC* contemporaneous hereditary breast cancer, *NCHBC* non-contemporaneous hereditary breast cancer

*ER/PR/HER-2*, (−): less than 1% staining, (+): 1% ~ 30% staining, (++): 30% ~ 80% staining, (+++): more than 80% staining, for *HER-2*, (±)Fish negative, *HER-2* gene non-amplification, (+) Fish positive, *HER-2* gene amplification; for *Ki-67*: (−): less than 14% staining, (+): 15% ~ 25% staining, (++): 25% ~ 50% staining, (+++): more than 50% staining

**Table 6:** Comparison of enzyme-labeled staining for *ER*, *PR* across subgroups

Variable	NHBC	HBC			Total
		CHBC	NCHBC	CHBC + NCHBC	
Expression of *ER*, n	152	18	20	38	190
(−)	44	2	2	4	48
(+)(++)(+++)	108	16	18	34	142
	*X*^2^ = 0.43 *P *> 0.05	*X*^2^ = 4.35 *P > 0.05	*X*^2^ = 3.25 **P > 0.05	*X*^2^ = 1.01 ^#^*P *> 0.05	
(−)(+)	66	3	7	10	76
(++)(+++)	86	15	13	28	114
	*X*^2^ = 3.71 *P *> 0.05	*X*^2^ = *3*.73 *P > 0.05	*X*^2^ = 0.51 **P > 0.05	*X*^2^ = 0.83 ^#^*P *> 0.05	
Expression of *PR*, n					
(−)	53	2	0	2	55
(+)(++)(+++)	99	16	20	36	135
	*X*^2^ = 13.38 *P *< 0.005	*X*^2^ = 5.93 *P > 0.05	*X*^2^ = 0.23 **P > 0.05	*X*^2^ = 3.7 ^#^*P *> 0.05	
(−)(+)	78	3	8	11	89
(++)(+++)	74	15	12	27	101
	*X*^2^ = 6.11 *P *< 0.05	*X*^2^ = 6.42 *P < 0.05	*X*^2^ = 0.91 **P > 0.05	*X*^2^ = 1.5 ^#^*P *> 0.05	

*HBC* hereditary breast cancer, *NHBC* non-hereditary breast cancer, *CHBC* contemporaneous hereditary breast cancer, *NCHBC* non-contemporaneous hereditary breast cancer

*ER/PR*, (−): less than 1% staining, (+): 1–30% staining, (++): 30–80% staining, (+++): more than 80% staining. *P* = NHBC versus HBC, *P = NHBC versus CHBC, **P = NHBC versus NCHBC, ^#^*P* = CHBC versus NCHBC

**Table 7:** Comparison of enzyme-labeled staining for *HER*-*2* and *Ki*-*67* across subgroups

Variable	NHBC	HBC			Total
		CHBC	NCHBC	CHBC + NCHBC	
Expression of *HER-2*, n	152	18	20	38	190
(−)(±)	88	8	11	19	107
(+)(++) (+++)	64	10	9	19	83
	*X*^2^ = 0.77 *P *> 0.05	*X*^2^ = 1.18 *P > 0.05	*X*^2^ = 0.06 **P > 0.05	*X*^2^ = 0.45 ^#^*P *> 0.05	
(−)(±)(+)	104	10	14	24	128
(++)(+++)	48	8	6	14	62
	*X*^2^ = 0.38 *P *> 0.05	*X*^2^ = 1.21 *P > 0.05	*X*^2^ = 0.02 **P > 0.05	*X*^2^ = 0.85 ^#^*P *> 0.05	
(−)(±)(+)(++)	144	13	18	31	175
(+++)	8	5	2	7	15
	*X*^2^ = 7.24 *P *< 0.01	*X*^2^ = 11.55 *P < 0.001	*X*^2^ = 0.72 **P > 0.05	*X*^2^ = 0.99 ^#^*P *> 0.05	
Expression of *Ki-67*, n					
(−)	36	6	7	13	49
(+)(++)(+++)	116	12	13	25	141
	*X*^2^ = 3.07 *P *> 0.05	*X*^2^ = *2*.46 *P > 0.05	*X*^2^ = 1.23 **P > 0.05	*X*^2^ = 0.42 ^#^*P *> 0.05	
(−)(+)	72	8	10	18	90
(++) (+++)	80	10	10	20	100
	*X*^2^ = 1.76 *P *> 0.05	*X*^2^ = *0*.81 *P > 0.05	*X*^2^ = 1.2 **P > 0.05	*X*^2^ = 0.01 ^#^*P *> 0.05	

*HBC* hereditary breast cancer, *NHBC* non-hereditary breast cancer, *CHBC* contemporaneous hereditary breast cancer, *NCHBC* non-contemporaneous hereditary breast cancer

*HER-2*, (−): less than 1% staining, (+): 1–30% staining, (++): 30–80% staining, (+++): more than 80% staining, (±) fish negative, *HER-2* gene non-amplification, (+) fish positive, *HER-2* gene amplification; for *Ki-67*: (−): less than 14% staining, (+): 15–25% staining, (++): 25–50% staining, (+++): more than 50% staining. *P* = NHBC versus HBC, *P = NHBC versus CHBC, **P = NHBC versus NCHBC, ^#^*P* = CHBC versus NCHBC

Meanwhile, the incidence rate of molecular phenotype classifications (Luminal A, Luminal B, *HER-2* positive, triple-negative) on breast cancer was also conducted across this study's subgroups (Table 5). Among all the included breast cancer cases, the incidence rate of *HER-2* positive was the highest (50%, 95/190), followed by Luminal A (34.74%, 66/190) and triple-negative (7.89%, 15/190), Luminal B was the lowest (7.37%, 14/190). In CHBC, the incidence rate of *HER-2* positive was also the highest (55.56%, 10/18), followed by Luminal A (27.78%, 5/18) and Luminal B (16.66%, 3/18); the triple-negative was not observed in this subgroup. In NCHBC, the incidence rate of Luminal A was the highest (50%, 10/20), followed by *HER-2* positive (45%, 9/20) and Luminal B (5%, 1/20); the triple-negative was not observed in this subgroup. In NHBC, the incidence rate of *HER-2* positive was the highest (50%, 76/152), followed by Luminal A (33.55%, 51/152) and triple-negative (9.87%, 15/152), Luminal B was the lowest (6.58%, 10/152). There were no significant differences in molecular phenotype classifications across the subgroups (*P* > 0.05).

### Expression of *Cytokeratin5/6* (*CK5/6*)*, E-cadhenrin* (*E-cad*)*, P63, and P120* across the breast cancer subgroups

We next compared the expression levels of other molecular markers in the breast cancer subgroups with different backgrounds (Table 8). As for *CK5/6*, there were no significant differences in expression levels across the breast cancer subgroups with different backgrounds (*P* > 0.05; Table 8). For *E-cad*, there was a significant difference in expression level between hereditary breast cancer patients and the non-hereditary breast cancer patients (*X*^*2*^ = 11.15, *P* < 0.005), as well as between CHBC and non-hereditary breast cancer patients (*X*^*2*^ = 9.75, *P* < 0.005). A significant difference was also observed in the expression level of *E-cad* between CHBC and non-hereditary breast cancer patients (*X*^*2*^ = 4.12, *P* < 0.05); however, there was no significant difference in the expression level of *E-cad* between CHBC and NCHBC (*P* > 0.05; Table 8). The expression of *P63* was significantly different only between CHBC patients and non-hereditary breast cancer patients (*X*^*2*^ = 4.49, *P* < 0.05); there were no significant differences among the other subgroups (*P* > 0.05; Table 8). Although negative expression of *P120* was not observed in NCHBC patients, no significant differences were found with respect to the expression of *P120* among the other subgroups (*P* > 0.05; Table 8).

**Table 8:** Comparison of enzyme-labeled staining for *CK5/6*, *E*-*cad*, *P63* and *P120* across subgroups

Variable	NHBC	HBC			Total
		CHBC	NCHBC	CHBC + NCHBC	
Expression of *CK5/6*, n	152	18	20	38	190
(−)	123	13	18	31	154
(+)	29	5	2	7	36
	*X*^2^ = 0.11 *P *> 0.05	*X*^2^ = 0.76 *P > 0.05	*X*^2^ = 0.47 **P > 0.05	*X*^2^ = 0.99 ^#^*P *> 0.05	
Expression of *E*-*cad*, n					
(−)	4	4	3	7	11
(+)	148	14	17	31	179
	*X*^2^ = 11.15 *P *< 0.005	*X*^2^ = 9.75 *P < 0.005	*X*^2^ = 4.12 **P < 0.05	*X*^2^ = 0.27 ^#^*P *> 0.05	
Expression of *P63*, n					
(−)	125	11	17	28	153
(+)	27	7	3	10	37
	*X*^2^ = 1.42 *P *> 0.05	*X*^2^ = 4.49 *P < 0.05	*X*^2^ = 0.00 **P > 0.05	*X*^2^ = 1.69 ^#^*P *> 0.05	
Expression of *P120*, n					
(−)	5	1	0	1	6
(+)	147	17	20	37	184
	*X*^2^ = 0.11 *P *> 0.05	*X*^2^ = 0.33 *P > 0.05			

*HBC* hereditary breast cancer, *NHBC* non-hereditary breast cancer, *CHBC* contemporaneous hereditary breast cancer, *NCHBC* non-contemporaneous hereditary breast cancer

*P* = NHBC versus HBC, *P = NHBC versus CHBC, **P = NHBC versus NCHBC, ^#^*P* = CHBC versus NCHBC

## Discussion

Recently, the incidence of breast cancer in female patients has been increasing each year. The latest cancer burden data released by the World Health Organization International Agency for Research on Cancer in 2020 shows that breast cancer has become the most common cancer in the world. As the most common malignant tumor, breast cancer is a serious threat to women's health. Although the pathogenic factors of breast cancer are diversified, clinical observation and basic research suggest that hereditary factors play an important role in the high-risk factors of breast cancer [3, 9] and that breast cancer with a hereditary family background is more likely to develop early. We found that some clinical parameters, such as age, differed between congenital breast cancer patients and overall breast cancer patients from preliminary clinical observations. Our study focused on a subpopulation of breast cancer patients, namely contemporaneous hereditary breast cancer (CHBC) patients. Studies on this subpopulation are rarely mentioned or reported in diagnosis and treatment guidelines or the literature [6, 7, 10]; therefore, it is necessary to conduct further research on this subpopulation.

In our study, clinical pathological tumor types, age, tumor location, histological grade, lymph node metastasis, and molecular phenotype expression (*ER, PR, HER-2, Ki-67, CK5/6, E-cad, P63,* and *P120*) for breast cancer patients were compared across the subgroups to discuss in detail the molecular phenotypes and clinical characteristics of CHBC compared to other types of breast cancer.

In this study, the age of onset observed for hereditary breast cancer patients was less than overall breast cancer patients, suggesting that age is a risk factor for breast cancer, which has also been reported in previous literature [11, 12]. Breast cancer patients with a contemporary hereditary background develop cancer earlier and are more likely to experience rapid disease progression, suggesting that the age factor plays a more significant role in this subpopulation. There may be an inherited genetic component related to the risk of early-onset breast cancer. For example, *BRCA1/2* and *P53* gene mutations may differ for the age of onset [13–15]. However, due to the small sample size collected in this study, the possible changes in the hereditary information of these patients must be further confirmed by future studies. However, the data on the BRCA gene mutation was insufficient in numbers to be analyzed in the present study and therefore was not included in the analysis. Nevertheless, the BRCA gene mutation is a substantial risk factor for breast cancer and warrants future comprehensive research. Meanwhile, considering that some CHBC patients presented with breast cancer in their child-bearing age, breast-conserving surgery should be adopted as far as possible during surgical treatment to retain the lactation capacity of the breast for breast-feeding infants [16].

Upon analysis of tumor location, there were no significant differences among the breast cancer subgroups. The incidence of breast cancer was equal on the left and right anatomic sides, indicating that anatomical site was not an influencing factor in the incidence of breast cancer. When we analyzed the pathological types in the breast cancer subgroups, we found that invasive ductal carcinoma was dominant in all subgroups, including patients with contemporaneous hereditary backgrounds (Table 1). The incidence of invasive ductal carcinoma was the lowest when comparing CHBC with the other subgroups, but this difference was insignificant (*P* > 0.05). The proportions of various pathological types in hereditary breast cancer and non-hereditary breast cancer were similar (Table 1). Some pathological types were missing in specific subgroups, resulting from the small sample size of this study. However, the proportions of major pathological types in each subgroup showed a similar trend. Upon completing a comprehensive analysis and comparing the proportion of pathological types among the subgroups with different backgrounds, no significant differences were observed (*P* > 0.05; Table 3). This suggests that hereditary factors may not affect the incidence of pathological types of breast cancer.

Upon comparing tissue differentiation classifications across the subgroups, we found that for the breast cancer group, which included CHBC patients, the proportion of grade II (moderate differentiation/middle level) was highest compared with the other subgroups (Table 3). Although the highest percentage (66.67%) of grade II was observed in CHBC, there were no significant differences when compared with the other subgroups (*P* > 0.05). The incidence percentage of grade III (poor differentiation/low level) was the highest (50%) in NCHBC, which may have been caused by the small sample size of this study, and requires further analysis with expanded sample size. The cell differentiation grade proportion of hereditary and non-hereditary breast cancer was similar (Table 1); all subgroups contained grade I, grade II, and III. Cell differentiation levels and proportion trends were consistent among the subgroups. After a comprehensive analysis and comparison of the composition ratio of cell differentiation grade across all the subgroups, no significant differences were found across all the subgroups (*P* > 0.05; Table 3), suggesting that hereditary factors may not affect the incidence of the differentiation grades of breast cancer cells.

The proportions of patients with lymph node metastasis in the breast cancer surgery population were compared across different hereditary backgrounds in this study. The results suggesting that hereditary factors may encourage a higher incidence of lymph node metastasis in breast cancer patients with hereditary backgrounds. However, further studies with expanded sample size are required to determine whether hereditary factors affects lymph node metastasis in CHBC.

Molecular phenotypic markers of breast cancer are often used to analyze breast cancer's biological behavior and select a subsequent treatment plan. This study explored the expression of several common breast cancer markers (*ER, PR, HER-2, Ki-67, CK5/6, E-cad, P63,* and *P120*) to search for possible molecular phenotypic changes in breast cancer patients across different backgrounds. The results showed that the expression levels of *ER* across the various subgroups of breast cancer patients may not be affected by hereditary factors. There was no significant difference in *PR* expression between CHBC patients and NCHBC patients. Hormone receptors are the targets of endocrine drug therapies for breast cancer [17, 18], and endocrine drugs alone or combined with other medications could significantly benefit hormone-dependent breast cancer patients with *ER/PR*-positive tumors [19, 20]. Moreover, there is evidence that endocrine drugs do not increase the risk of other tumors in breast cancer patients [21]. From the results of hormone receptor expression, although the expected therapeutic effects of hormone receptor-targeting endocrine drugs may not be significantly different from those of other breast cancer patients, endocrine drugs may also benefit estrogen receptor-positive CHBC patients.

*HER-2* expression was used to evaluate patients for possible response and efficacy of drugs that target *HER-2*. For patients with positive or high *HER-2* expression, targeted drugs after surgery and chemotherapy could bring significant benefits [22, 23]. Patients with advanced breast cancer could benefit significantly from targeted drugs [24, 25]. In this study, we observed a substantial difference in the expression of *HER-2* marker between hereditary and non-hereditary breast cancer patients (*P* < 0.05); Based on the results of the further analysis, we speculated that patients with hereditary breast cancer, especially those with CHBC, may respond better to *HER-2-*targeted drugs.

*Ki-67* is a cell proliferation antigen marker, reflecting the state of cell proliferation and rapidly and reliably reflecting the proliferation rate of malignant tumors, which is related to the development, metastasis, and prognosis of various malignant tumors [26]. This study suggests that *Ki-67* expression might not be affected by hereditary factors [27]. CHBC patients might not differ significantly from other breast cancer patients in their expected treatment response to chemotherapy agents targeting *Ki-67*. In addition, combined with the conclusion that there was no significant difference (*P* > 0.05) in the composition ratio of cell differentiation grade in each subgroup, we found that the degree of cell differentiation observed from cell morphology was consistent with the degree of cell proliferation confirmed from the perspective of epigenetics. Additionally, the expression of *Ki-67* was consistent with the degree of cancer cell differentiation.

Molecular phenotype classification of breast cancer plays an essential role in guiding the comprehensive treatment and prognosis of breast cancer [28, 29]. This study performed subgroup analyses of the four molecular phenotypes (Luminal A, Luminal B, *HER-2* positive, triple-negative). The results demonstrated that CHBC patients might be more suitable for targeted drug therapy due to the high proportion of *HER-2* positive (55.56%, 10/18). At the same time, some patients may also benefit from endocrine drug therapy due to the expression of hormone receptors.

The expression of other common breast cancer markers (*CK5/6, E-cad, P63,* and *P120*) was also analyzed in this study. Molecular typing of breast cancer is closely related to the prognosis of patients. Breast cancer with positive expression of *CK5/6* has a poor prognosis [30, 31]. In addition, breast cancer with positive expression of *CK5/6* may be prone to invasion and metastasis due to epithelial-mesenchymal transformation due to loss of *E-cad* expression [32]. As a metastatic suppressor, *E-cad* plays a vital role in maintaining high adhesion characteristics and inhibiting metastasis invasion of cancer cells. Previous studies showed that E-cad's high expression loss was an independent factor for poor breast cancer prognosis [31, 33]. As a *P53* tumor suppressor gene family member, *P63* also plays an important role in the occurrence, development, invasion, and metastasis of tumors [31, 34]. *P120* is an intracellular signal transduction and cell adhesion molecule that binds to the intracellular segment near the membrane end of the *E-cad* and regulates *E-cad* mediated intracellular signal transduction and cell adhesion. *P120* plays an important role in the process of mediating cell adhesion, as well as tumor occurrence and development [35].

In this study, the expression of aforementioned breast cancer markers were observed across the disease subgroups. We observed no significant difference in the expression level of *CK5/6* in breast cancer patients with different backgrounds (*P* > 0.05), suggesting that the expression of *CK5/6* might not be affected by hereditary factors. The expression level of *CK5/6* might not be the factor leading to the difference between hereditary breast cancer and non-hereditary breast cancer. For the *E-cad* marker, significant differences were observed between hereditary breast cancer patients and non-hereditary breast cancer patients (*P* < 0.005), between CHBC patients and non-hereditary breast cancer patients (*P* < 0.005), and also between NCHBC patients and non-hereditary breast cancer patients (*P* < 0.05). There was no significant difference in the expression level of *E-cad* between the CHBC patients and NCHBC patients (*P* > 0.05), suggesting that the *E-cad* expression level might be influenced by hereditary factors. High expression loss of *E-cad* was observed in hereditary breast cancer patients, and the prognosis of hereditary breast cancer patients was worse than that of non-hereditary breast cancer patients. Although there was no significant difference in the expression level of *E-cad* between the CHBC patients and NCHBC patients, the former had higher expression levels of *E-cad*, suggesting that the prognosis of CHBC patients may be worse than other breast cancer subgroups. For *P63*, a significant difference was observed only between CHBC patients and non-hereditary breast cancer patients (*P* < 0.05), suggesting that the loss of *P63* expression might lead to CHBC that is different from other subgroups and acts an independent risk factor of breast cancer. There were no significant differences in the expression levels of *P120* among all the subgroups (*P* > 0.05), suggesting that the expression level of *P120* might not be affected by hereditary factors, and might not be the factor leading to the difference in the incidence of hereditary breast cancer and non-hereditary breast cancer.

Originating from a single-center, the clinical breast cancer observation samples in this study were limited; the number of observation subpopulations was particularly small. Although we identified significant differences between some of the subgroups in our study, future studies with expanded sample size are required to confirm our findings.

## Conclusion

Family inheritance is an important factor in the development of breast cancer, and the contemporaneous hereditary breast cancer (CHBC) accounts for 9.47% (18/190) of the breast cancer surgery population. CHBC patients showed earlier age of onset, with additional differences in molecular phenotype and clinical characteristics between CHBC and non-hereditary breast cancer patients. Therefore, these patients should receive paid opportunities to volunteer for clinical work.

## Data Availability

The datasets used and analysed during the current study are available from the corresponding author on reasonable request.
